# Effects of Work Engagement and Barriers on Evidence-Based Practice Implementation for Clinical Nurses: A Cross-Sectional Study

**DOI:** 10.3390/healthcare12222223

**Published:** 2024-11-07

**Authors:** Kijung Mun, Minsung Lee, Jaelan Shim

**Affiliations:** College of Nursing, Dongguk University-Wise, Gyeongju 38066, Republic of Korea

**Keywords:** work engagement, evidence-based practice barriers, evidence-based practice implementation

## Abstract

**Background/Objectives**: Implementing evidence-based practice (EBP) among healthcare professionals is a vital strategy for improving the quality of healthcare services, patient outcomes, and professional role satisfaction. In this study, we aimed to identify the effects of work engagement and barriers to EBP implementation among clinical nurses. **Methods**: In this cross-section study, we collected data from 184 nurses with at least 1 year of clinical experience using a questionnaire. The study was performed in three general hospitals in Korea between 17 July and 4 August 2023. Following data collection, we performed descriptive statistics, independent sample *t*-test, one-way analysis of variance, Scheffe’s post hoc test, Pearson’s correlation analysis, and multiple regression to analyze the data. **Results**: EBP implementation was found to be positively correlated with work engagement (*r* = 0.32, *p* < 0.001) and negatively correlated with barriers to implementing EBP (*r* = −0.44, *p* < 0.001). Factors influencing EBP implementation included work engagement (β = 0.14, *p* = 0.041) and barriers (β = −0.39, *p* < 0.001). Notably, barriers had a greater influence on EBP implementation than work engagement. **Conclusions**: Policy support, including performance incentives and training policies, among others, along with an organizational environment that provides necessary resources, should be established to encourage nurses’ engagement, which influences their organizational performance to improve EBP. In addition, it is crucial to develop and strengthen educational and support programs for nurses regarding EBP to help identify and minimize barriers to implementing this practice.

## 1. Introduction

Evidence-based practice (EBP) involves a series of processes that influence decision-making based on the latest scientific evidence and findings. It entails integrating the clinical expertise of nurses and considering patient preferences within the healthcare context and available resources [1]. EBP is a holistic approach that integrates knowledge, skills, and attitudes that healthcare professionals acquire and learn throughout their lives, which enhances the quality of their practice, distinguishes appropriate evidence, and suggests ideal standards for providing safe practice, especially in nursing [2]. Applying EBP can help reduce healthcare costs [1,3], and when EPB is properly used in the nursing practice setting, it provides the best possible care leading to positive patient outcomes [4]. Accordingly, the importance of applying this practice in nursing is gradually increasing.

According to a previous study, EBP is not being applied properly due to barriers, including organizational, communication-related, nursing, and research factors [5].

Firstly, organizational factors include the lack of support, such as time and finance, and the system for providing up-to-date, evidence-based nursing; further organizational factors include low accessibility to evidence, such as the lack of opportunity to participate in research, the reduced autonomy of nursing organization, strict organizational culture, and various environmental constraints [6]. Secondly, there are the communication-related factors of EBP, including the lack of communication, mutual understanding, and cooperation among medical professionals [7]. Thirdly, nursing factors include the lack of overall knowledge among nurses regarding initiating research-related activities or utilizing research results and low self-confidence and motivation [8,9]. Finally, research factors include the lack of high-quality clinical studies, difficulty in controlling subjects and variables in clinical studies, and difficulty with utilizing research results and clinical practice [8,9]. In particular, a lack of organizational support for education and research is associated with lower EBP implementation, indicating the need for policies to strengthen the implementation of this practice at the organizational level.

Moreover, work engagement is the motivational and psychological state of having a positive and fulfilling attitude toward work [10]. In professional nursing practice, it is an important factor that should be considered when healthcare systems attempt to address major challenges, such as global nursing shortage, pressure to reduce healthcare costs, increasing demand for high-quality care, and positive patient outcomes [11]. Furthermore, the members of an organization who have high work engagement are more energetic, and they enthusiastically commit themselves to work. Thus, work engagement can be viewed as an important factor of organizational culture for improving work performance and satisfaction [12]. Findings from a previous study revealed that work engagement significantly mediates the relationship between organizational support for EBP and educational growth and perceived professionalism [13].

In addition, previous studies identified an association between work engagement and turnover intention among nurses [14,15]. Nurses with high work engagement were reported to have higher job implementations and lower turnover intentions [16]. Additionally, these nurses tend to enjoy and happily commit themselves to their work, while performing duties that cover a broader scope. Moreover, they extend their activities to help and collaborate with colleagues to benefit the organization. This feature can be viewed as a factor necessary for applying EBP that positively influences the achievement of organizational goals [17].

Notably, work engagement brings a high level of energy and enthusiasm to nurses, which can influence the achievement of personal and organizational goals [18].

Studies on EBP have been conducted [19,20,21,22]; however, no studies have identified the correlations between the implementation of EBP and barriers and work engagement that can enhance organizational performance and professionalism. Therefore, this study aimed to identify the correlations between work engagement, barriers, and the application of EBP in clinical nurses and the effects of work engagement and barriers on the application of EBP to provide basic data for nursing an intervention that can improve the application of EBP in the future.

## 2. Materials and Methods

### 2.1. Study Design

This was a cross-sectional study.

### 2.2. Study Participants

We included nurses working at three general hospitals in Korea in this study. The inclusion criteria were as follows:

(1) Nurses with at least 1 year of experience at the same hospital [21];
(2) Nurses responsible for direct nursing duties in a general hospital;
(3) Nurses who understood the purpose of this study and voluntarily agreed to participate in the survey;
(4) Nurses that could read, write, and speak Korean.

We excluded nurses with <1 year of clinical experience because they were expected to have difficulty in making evidence-based decisions and a lack awareness of evidence-based practice due to lack of work adaptation and clinical experience [21].

We calculated the sample size using G Power version 3.1.9.2. We performed multiple regression analysis, and the sample size was calculated to be 184 with a significance level of 0.05, moderate effect size of 0.15, statistical power of 0.95, and 12 predictors (sex, age, marital status, highest education level, current position, work department, total clinical experience, work type, need for EBP, EBP training experience, work engagement, and barriers). Considering a 10% dropout rate, we distributed 205 sets of questionnaires. Questionnaires with missing or incomplete responses were identified, and missing data were excluded from the analysis sample, with a final sample of 184 questionnaires used for statistical analysis.

### 2.3. Study Instruments

#### 2.3.1. EBP Implementation

We measured the implementation of EBP using a version of the Evidence-Based Practice Questionnaire, a self-reporting scale originally developed by Upton and Upton (2006) [23]. The questionnaire was subsequently translated into Korean and its reliability and validity have been verified by Lim et al. (2011) [24] to identify the implementation of EBP among nurses and used in a preliminary survey. The instrument comprised 24 items in three domains: EBP knowledge (*n* = 6), attitude (*n* = 4), and use (*n* = 14). It is a tool whose validity has been verified using convergent and discriminant validity. Each item was rated on a 7-point Likert scale, with higher scores indicating greater use of EBP, more positive attitude toward EBP implementation, and better knowledge of EBP implementation. Furthermore, the reliabilities (Cronbach’s α) of the entire scale and knowledge, attitude, and use domains were 0.87, 0.85, 0.79, and 0.91, respectively, in the study by Upton and Upton (2006) [23] and 0.87, 0.88, 0.80, and 0.90 in this study, respectively.

#### 2.3.2. Barriers to Implementing EBP

Barriers to implementing EBP are the perceived barriers to using research evidence, which is a key element of EBP. We measured the barriers using an instrument originally developed by Thompson et al. (2004) [25] and subsequently translated into Korean by Lee and Park (2011) [8]. The instrument comprised 29 items in four domains: organizational (7 items), communication-related (7 items), nursing (8 items), and research (7 items) factors. Each item was rated on a 5-point Likert scale with higher scores indicating higher levels of perceived barriers. The reliability (Cronbach’s α) of the instrument was 0.83 in the study by Thompson et al. (2004) [25] and 0.94 in this study.

#### 2.3.3. Work Engagement

We measured work engagement using a version of the Utrecht Work Engagement Scale originally developed by Schaufeli and Bakker (2003) [26] and subsequently translated into Korean, revised, and supplemented by Kim (2015) [27]. This scale comprised 17 items in three subscales: vigor (6 items), dedication (5 items), and absorption (6 items). Each item was rated on a 5-point Likert scale with higher scores indicating higher levels of work engagement. The reliability (Cronbach’s α) of this scale was 0.88 in this study.

## 3. Data Collection and Analyses

### 3.1. Data Collection

In this study, we collected the data between 17 July and 4 August 2023. The data collection method involved the researcher personally visiting hospitals to distribute and retrieve questionnaires after obtaining permission from the manager of each ward. The purpose and method of this study and the guarantee of anonymity and autonomy for research participation were explained to the participants. Additionally, possible benefits and disadvantages and the possibility of giving up the survey during the research were also directly explained to the participants. Written consent for research participation was obtained from those who voluntarily agreed to participate. In addition, an incentive was provided for participants as appreciation after completing the survey, and all data were kept safely in an envelope to prevent damage due to external exposure.

The content validity and internal reliability of the questionnaire were assessed using the Cronbach alpha coefficient. The Cronbach alpha coefficient ranged from 0.79 to 0.94. The reliability and validity of this research tool were examined according to the criteria where a value of 0.7 is considered acceptable and 0.9 is considered very good [28]. The supervisor and investigator closely supervised the performance of the data collectors for the actual data collection. The data were rechecked after data collection for completeness and internal validity.

### 3.2. Data Analyses

We analyzed the collected data using the IBM SPSS 27.0 program (IBM Corp, Armonk, NY, USA). The specific analysis methods were as follows:

- The differences in work engagement, barriers to EBP, and EBP implementation according to the general and job-related characteristics were tested using the *t*-test and analysis of variance at a significance level of *p* < 0.05, whereas Scheffe’s test was used as the post hoc test.
- The correlations between work engagement, barriers, and EBP implementation were analyzed using Pearson’s correlation coefficients.
- The effects of work engagement and barriers on the implementation of EBP were analyzed using multiple regression analysis.
- The reliability of the instruments used was determined using Cronbach’s α.

## 4. Ethical Consideration

In this study, we started collecting data after receiving approval from the Institutional Review Board of the Dongguk University WISE campus of Korea (DGU IRB 20230010), and the research procedures were conducted following the Helsinki Principles of Research Ethics revised in 2013.

## 5. Results

### 5.1. Differences in Work Engagement, Barriers to Implementing EBP, and EBP Implementation According to General and Job-Related Characteristics

Differences in work engagement, barriers to implementing EBP, and EBP implementation according to the general and job-related characteristics are shown in Table 1.

Work engagement significantly differed regarding age (F = 4.68, *p* < 0.001), marital status (*t* = −4.06, *p* < 0.001), highest education level (F = 10.60, *p* < 0.001), current position (F = 13.43, *p* < 0.001), total clinical experience (F = 4.41, *p* = 0.005), work type (*t* = −3.68. *p* < 0.001), and the need for EBP (F = 5.62, *p* = 0.004).

Furthermore, barriers to implementing EBP significantly differed regarding sex (*t* = 2.45, *p* = 0.015), need for EBP (F = 4.39, *p* = 0.014), and EBP training experience (*t* = −2.08, *p* = 0.039).

In addition, EBP implementation significantly differed regarding sex (*t* = −2.56, *p* = 0.011), age (F = 4.65, *p* = 0.004), marital status (*t* = −3.28, *p* = 0.001), highest education level (F = 5.39, *p* = 0.005), current position (F = 9.38, *p* < 0.001), and work type (*t* = −2.08, *p* = 0.039).

### 5.2. Level of Work Engagement, Barriers to Implementing EBP, and EBP Implementation

The levels of study variables, including sub-dimensions, are shown in Table 2.

### 5.3. Correlations Between Work Engagement, Barriers to Implementing EBP, and EBP Implementation

Results from the correlation analyses between work engagement, barriers to implementing EBP, and EBP implementation are shown in Table 3.

EBP implementation was positively and negatively correlated with work engagement (r = 0.3.2, *p* < 0.001) and barriers to implementing EBP (r = −0.44, *p* < 0.001), respectively. Moreover, work engagement was negatively correlated with the barriers to implementing EBP (r = −0.21, *p* = 0.005).

### 5.4. Effects of Work Engagement and Barriers on the Implementation of EBP

The results from multiple regression analysis performed to identify the effects of work engagement and barriers on the implementation of EBP are shown in Table 4.

We tested whether the assumptions of the regression analysis were satisfied before performing the regression analysis. The results showed a Durbin–Watson statistic score of 2.03, a value close to 2, indicating no autocorrelation. The tolerance limit (0.33–0.98) was ≥0.1, and the variance inflation factor (1.02–3.06) did not exceed the cutoff of 10, indicating no multicollinearity issues among the variables. Hence, the assumptions of the regression analysis were satisfied.

We performed regression analysis to determine the effects of significant variables identified in the univariate analysis of differences in EBP implementation based on the general and job-related characteristics (sex, age, marital status, highest education level, current position, and work type) and the independent variables of this study (work engagement and barriers to implementing EBP). The analysis revealed that the variable with the greatest influence on EBP implementation was barriers to implementing EBP (β = −0.39, *p* < 0.001), followed by work engagement (β = 0.14, *p* = 0.041). The explanatory power of the modified model was 28.0% (Adj R^2^ = 0.28, *p* < 0.001).

## 6. Discussion

In this study, we aimed to identify the correlations between work engagement, barriers, and EBP implementation among clinical nurses and the effects of work engagement and barriers to implementing EBP to provide basic data for interventional studies that can improve its use among nurses. Work engagement and barriers to implementing EBP were predictors of the implementation of EBP, and barriers had a greater influence on the EBP implementation than work engagement.

The barrier level to implementing EBP among the participants was 2.82 (±0.52) of 5 points, indicating a moderate level. Regarding the four domains (communication-related, organizational, nursing, and research factors), organizational factors were identified as the greatest barriers to implementing EBP with a score of 3.07 (±0.62) points. In a multicenter randomized stratified study on EPB barriers and facilitators among 100 nurses, organizational factors were categorized into the lack of either EBP resources, support, manpower, interest, and work overload [29]. In addition, nurses recognized the organizational need to fully accept the atmosphere for supporting infrastructure and successfully implementing EBP [30], confirming the need for active efforts to instigate changes within the organization for the implementation of EBP.

Based on the domains of barriers to implementing EBP, organizational factors showed the highest score (3.07 points), followed by communication-related (3.04 points), research (2.97 points), and nursing (2.88 points) factors. Common organizational barriers include time constraints, access to sufficient staff, EBP, and research resources, inadequate training, lack of technology, funding, and organizational support to address knowledge gaps. Other disenfranchising factors included isolation from professionals, workload, and the inability to appropriately assess the quality of evidence and implement new knowledge into practice [31,32]. It is believed that these organizational barriers may have prevented nurses from implementing EBP and changing practices within their organizations.

Furthermore, the mean work engagement score was 3.08 out of 5 points. Overseas studies that used the same scale as the present study reported higher scores, including a study in Saudi Arabia by Alluhaybi et al. (2024) [33] that measured the association between leadership and work engagement among 450 clinical nurses. They reported a mean score of 4.03 points. Moreover, a study in the Netherlands by Penturij-Kloks et al. (2023) [34] compared work engagement among 1697 nurses before and after the COVID-19 pandemic and reported a mean score of 4.28 points.

These differences can be attributed to each country having different levels of motivational factors, incentives, and autonomy in work decisions that significantly influence work engagement among nurses [33]. Furthermore, such factors may be lower in Korea than in other countries.

In addition, nurses across the United States showed a lower turnover intention when they had higher work engagement. Notably, work engagement was positively associated with job satisfaction and the perceived quality of care [14]. Therefore, nursing managers should continuously monitor the work engagement of nurses and establish strategies for enhancing the nursing environment to increase the organizational commitment and performance.

Both studies in Korea and abroad reported that work engagement has a positive influence on organizational and nursing practice performance [35]; therefore, the importance of work engagement among nurses should be clearly recognized, and work engagement among members should be regularly measured. Furthermore, replication studies on this topic should continue to identify and apply the association between work engagement and nurse performance.

The mean implementation of the EBP score of the participants in this study was 4.18 out of 7 points, which was lower than the 4.85 points reported in a study by Wang et al. (2021) [35] for EBP implementation among 286 EBP mentors in six hospitals in China. Notably, 78.3% of the participants in this study recognized the need for EBP implementation; however, only 48.9% had completed EBP-related training, which explains why relatively lower EBP implementation was observed than that observed by Wang et al. Therefore, providing opportunities for more diverse EBP-related training is needed, and future studies should verify the effectiveness of educational interventions.

According to a systematic review of EBP among nurses [36], most nurses had a positive perception of EBP; however, they were unable to sufficiently apply EBP in clinical settings due to barriers, such as the lack of knowledge and training; moreover, academic education was a facilitator of EBP. These findings suggest that EBP facilitates nursing education and research and contributes to academic advancement to enhance learning and knowledge, which can improve the implementation of EBP among nurses.

Furthermore, the analysis of correlations between work engagement, barriers to implementing EBP, and EBP implementation among nurses in this study showed that higher work engagement was correlated with lower barriers and higher EBP implementation, and that lower barriers were correlated with higher EBP implementation. Hence, work engagement and barriers both have a significant influence on the EBP implementation, confirming that they are important factors in its implementation. Therefore, EBP can be strengthened in the field of nursing by overcoming barriers to implementing EBP through high work engagement and exploring measures for enhancing the quality of patient care. Future research and policy development should continue to strengthen the implementation of EBP among nurses.

### Strengths and Limitations of This Study

This study has some limitations. First, we included nurses from three general hospitals in Korea using a short data collection time period and the findings do not represent the characteristics of all medical institutions in Korea. Thus, caution is needed when generalizing the findings. Second, this study only examined the personal characteristics of nurses in association with work engagement, barriers, and EBP implementation; however, organizational variables, such as the attitude of managers and human resources, were not considered. Therefore, future multicenter, large-scale exploratory studies are needed. Finally, because this study was a descriptive survey with results derived from nurses using a self-reporting questionnaire, there are difficulties with causal inferences. Despite these limitations, our study findings were significant in that they identified the correlations between work engagement, barriers, and EBP implementation among nurses working in three general hospitals in Korea to present basic data for promoting and exploring organizational and personal measures for increasing EBP implementation among nurses.

## 7. Conclusions

Clinical nurses with higher work engagement have lower barriers to implementing EBP and higher EBP implementation than those with lower work engagement. Furthermore, maintaining and implementing EBP within an organization is itself a dynamic and ongoing process of knowledge creation. Therefore, it is important to create policies and an environment that can maintain and facilitate the work performance of nurses. In addition, efforts are needed to identify and resolve the barriers to implementing EBP within organizations and eventually strengthen this practice. Hence, EBP education and support programs should be developed to help nurses become familiar with this practice, and studies should be conducted to verify the effectiveness of such programs.

In this study, we utilized the STROBE statement—a checklist of items that should be included in the reports of cross-sectional studies [37], which can be found in the Supplementary Materials.

## Figures and Tables

**Table 1:** Differences in work engagement, barriers to implementing EBP, and EBP implementation according to general and job-related characteristics.

Variables	Categories	N (%) or Mean ± SD	Work Engagement		Barriers to Implementing EBP		Evidence-Based Practice Implementation	
			Mean ± SD	*t* or F (*p*) Scheffê	Mean ± SD	*t* or F (*p*)	Mean ± SD	*t* or F (*p*) Scheffê
Age †	<30 a	116 (63.0)	3.02 ± 0.46	4.68 (<0.001)	2.97 ± 0.46	0.40 (0.754)	4.08 ± 0.67	4.65 (0.004)
(y)	30–39 b	40 (21.7)	3.03 ± 0.53	a, b < c	3.03 ± 0.55		4.21 ± 0.51	a, b < c
	40–49 c	26 (14.1)	3.40 ± 0.47		2.98 ± 0.49		4.60 ± 0.77	
	≥50 d	2 (1.1)	3.12 ± 0.75		3.28 ± 0.34		4.41 ± 1.06	
		30.17 ± 6.62						
Sex	Male	21 (11.4)	3.06 ± 0.50	−1.61 (0.110)	3.74 ± 0.49	2.45 (0.015)	4.54 ± 0.51	−2.56 (0.011)
	Female	163 (88.6)	3.24 ± 0.34		3.02 ± 0.48		4.14 ± 0.69	
Marital status	Unmarried	50 (27.2)	3.31 ± 0.52	−4.06 (<0.001)	3.00 ± 0.52	−0.41 (0.681)	4.45 ± 0.69	−3.28 (0.001)
	Married	134 (72.8)	2.99 ± 0.45		2.98 ± 0.47		4.09 ± 0.65	
Educational level †	Associate degree a	46 (25.0)	2.87 ± 0.48	10.60 (<0.001)	3.05 ± 0.52	0.53 (0.591)	3.97 ± 0.79	5.39 (0.005)
	Bachelors b	136 (73.9)	3.13 ± 0.46	a, b < c *	2.96 ± 0.48		4.24 ± 0.61	a < c *
	≥Masters c	2 (1.1)	4.15 ± 0.71		2.91 ± 0.17		5.21 ± 0.06	
Position †	Staff nurse a	145 (78.8)	3.00 ± 0.47	13.43 (<0.001)	3.00 ± 0.47	2.74 (0.067)	4.08 ± 0.65	9.38 (<0.001)
	Charge nurse b	25 (13.6)	3.39 ± 0.38	a < b,c *	2.80 ± 0.57		4.66 ± 0.73	a < b *
	≥Nursing manager c	14 (7.6)	3.47 ± 0.47		3.12 ± 0.44		4.41 ± 0.52	
Working unit	Internal medicine unit	59 (32.1)	3.08 ± 0.48	1.25 (0.291)	3.11 ± 0.42	1.85 (0.122)	4.17 ± 0.67	1.64 (0.166)
	Surgical unit	81 (44.0)	3.04 ± 0.51		2.93 ± 0.42		4.18 ± 0.65	
	Special unit	33 (17.9)	3.15 ± 0.52		2.87 ± 0.68		4.32 ± 0.70	
	OPD	9 (4.9)	3.29 ± 0.27		3.09 ± 0.50		4.10 ± 0.82	
	Others	2 (1.1)	2.59 ± 0.00		3.00 ± 0.00		3.13 ± 0.00	
Clinical career †	1–<3 a	57 (31.0)	3.00 ± 0.42	4.41 (0.005)	2.85 ± 0.50	2.29 (0.079)	4.05 ± 0.68	2.35 (0.074)
(y)	3–<5 b	39 (21.2)	3.05 ± 0.42	a,c < d *	3.05 ± 0.50		4.14 ± 0.72	
	5–<10 c	42 (22.8)	2.97 ± 0.057		3.05 ± 0.47		4.17 ± 0.52	
	≥10 d	46 (25.0)	3.29 ± 0.50		3.04 ± 0.45		4.40 ± 0.74	
Types of work shift	Rotating shift	155 (84.2)	3.02 ± 0.49	−3.68 (<0.001)	2.98 ± 0.49	−0.14 (0.889)	4.14 ± 0.64	−2.08 (0.039)
	Fixed shift	29 (15.8)	3.38 ± 0.41		3.00 ± 0.48		4.42 ± 0.82	
Perception of EBP necessary †	Yes a	144 (78.3)	3.13 ± 0.50	5.62 (0.004)	2.95 ± 0.50	4.39 (0.014)	4.22 ± 0.67	1.10 (0.334)
	No b	2 (1.1)	2.41 ± 0.00	b < a	3.86 ± 0.00		4.00 ± 0.00	
	Unsure c	38 (20.7)	2.90 ± 0.40		3.07 ± 0.39	a,c < b	4.05 ± 0.73	
Experience of EBP education	Yes	90 (48.9)	3.08 ± 0.49	0.10 (0.921)	2.91 ± 0.48	−2.08 (0.039)	4.15 ± 0.71	−0.66 (0.512)
	No	94 (51.1)	3.07 ± 0.49		3.06 ± 0.49		4.22 ± 0.65	

EBP, evidence-based practice; OPD, out-patient department; SD, standard deviation. †, * = Post hoc analysis was performed using the Sheffe test. *p*-value (*p*) < 0.05 was considered statistically significant.

**Table 2:** Level of work engagement, barriers to implementing EBP, and EBP implementation (*n* = 184).

Variables	Categories	Item	Mean ± SD	Range
Work engagement		17	3.08 ± 0.49	1–5
	Vigor	6	2.76 ± 0.60	
	Dedication	5	3.54 ± 0.60	
	Absorption	6	3.01 ± 0.56	
Barriers to implementing EBP		29	2.82 ± 0.52	1–5
	Communication	7	3.04 ± 0.51	
	Organization	7	3.07 ± 0.62	
	Nursing	8	2.88 ± 0.55	
	Research	7	2.97 ± 0.57	
Evidence-based practice implementation		24	4.18 ± 0.68	1–7
	Knowledge	6	4.26 ± 1.09	
	Attitude	4	4.18 ± 0.92	
	Performance	14	4.15 ± 0.70	

SD, standard deviation.

**Table 3:** Correlations between work engagement, barriers to implementing EBP, and EBP implementation.

Variables	Work Engagement	Barriers to Implementing EBP	Evidence-Based Practice Implementation
	r (*p*)	r (*p*)	r (*p*)
Work engagement	1		
Barriers to implementing EBP	−0.21 (0.005)	1	
Evidence-based practice implementation	0.32 (<0.001)	−0.44 (<0.001)	1

**Table 4:** Effects of work engagement and barriers on the implementation of EBP.

Variables	B	SE	β	*t*	*p*
Age	0.01	0.01	0.14	1.27	0.206
Sex (Ref: Female)	−0.26	0.14	−0.012	−1.87	0.064
Marital status (Ref: Married)	−0.17	0.15	−0.11	−1.13	0.262
Education (Ref: ≥Master’s)	−0.45	0.46	−0.07	−0.99	0.323
Position (Ref: Staff nurse)	0.13	0.21	0.05	1.61	0.543
Types of work shift (Ref: Fixed shift)	−0.05	0.14	−0.03	−0.37	0.710
Work engagement	0.20	0.10	0.14	2.06	0.041
Barriers to implementing EBP	−0.55	0.09	−0.39	−6.01	<0.001
Model fit: R^2^ = 0.31, Adj. R^2^ = 0.28 F = 28.83, *p* < 0.001					

SE, standard error; Ref, reference. A *p*-value (*p*) < 0.05 was considered statistically significant.

## Data Availability

Data are contained within the article.

## Supplementary Materials

The following supporting information can be downloaded at: https://www.mdpi.com/article/10.3390/healthcare12222223/s1, STROBE Statement.
